# Diaphragmatic Mitochondrial Myopathy in a Patient-Derived Mouse Model of Barth Syndrome

**DOI:** 10.3390/jdb14030040

**Published:** 2026-09-03

**Authors:** Kristen Tentler, Paige L. Snider, Catalina Matias, Jeffrey J. Brault, Simon J. Conway

**Affiliations:** 1 Herman B. Wells Center for Pediatric Research, Department of Pediatrics, Indiana University School of Medicine, 1044 West Walnut Street, Indianapolis, IN 46202, USA; 2 Division of Pediatric Pulmonology, Allergy and Sleep Medicine, Department of Pediatrics, Indiana University School of Medicine, 705 Riley Hospital Drive, Indianapolis, IN 46202, USA; 3 Indiana Center for Musculoskeletal Health, Department of Anatomy, Cell Biology & Physiology, Indiana University School of Medicine, 635 Barnhill Drive, Indianapolis, IN 46202, USA

**Keywords:** Barth syndrome, tafazzin, diaphragmatic mitochondrial myopathy, CRISPR/Cas mouse model, bioenergetics

## Abstract

Barth syndrome (BTHS) is a rare, X-linked genetic disorder caused by mutations in the enzyme TAFAZZIN (TAZ), resulting in insufficient cardiolipin (CL) remodeling and mitochondrial dysfunction. While BTHS respiratory distress and breathing difficulties are commonly reported, the precise role of intrinsic respiratory tissue vulnerabilities has only recently begun to be appreciated. Historically, BTHS respiratory distress is frequently attributed to secondary consequences like cardiomyopathy or generalized skeletal myopathy, leaving the intrinsic vulnerability of vital respiratory muscles poorly understood. Using a patient-tailored point mutant knock-in mouse model (*Taz^PM^*) harboring a stable but enzymatically deficient Taz^D75H^ protein, we investigated the autonomous physiological and metabolic responses in the diaphragm and lungs. Contrary to the paradigm that respiratory muscles are unaffected, *Taz^PM^* diaphragms exhibit structurally abnormal mitochondria and undergo a survival-critical, bifurcated compensatory remodeling response to prevent fatal respiratory failure under severe bioenergetic stress. The *Taz^PM^* adaptive mechanism is orchestrated by chronic activation of the mitochondrial Integrated Stress Response (ISR) via the Gcn2/eIF2α signaling pathway. This stress pathway halts global translation to conserve cellular ATP at the expense of reduced NAD+ levels, while selectively upregulating defensive mitokines and metabolic sirtuins and structural muscle remodeling. Furthermore, the *Taz^PM^* diaphragm transitions into a highly specialized, slow-twitch motor system that is expected to reduce the energy cost per contraction. Concurrently, despite *Taz^PM^* lungs exhibiting structurally abnormal mitochondria, they resist generalized mitochondrial collapse despite ADP reduction, executing tissue-specific metabolic reprogramming and localized biochemical adaptations to sustain respiratory homeostasis.

## 1. Introduction

Barth syndrome (BTHS; OMIM #302060) is a rare, life-threatening, X-linked genetic disorder characterized by cardiomyopathy, skeletal muscle myopathy, neutropenia, and organic aciduria [1,2]. The disease is caused by loss-of-function mutations in the *TAFAZZIN* gene, which encodes TAFAZZIN, a highly conserved phospholipid transacylase indispensable for lipid metabolism and the structural integrity of the inner mitochondrial membrane (IMM) [3,4]. Under physiological conditions, TAZ catalyzes the final remodeling step of cardiolipin (CL), a unique dimeric phospholipid that orchestrates the assembly of electron transport chain (ETC) supercomplexes and stabilizes the IMM cristae curvature [4,5]. Consequently, dysfunctional and/or deficient TAZ impairs CL maturation, causing an accumulation of immature monolysocardiolipin (MLCL) and precipitating severe mitochondrial dysfunction alongside compromised cellular adenosine triphosphate (ATP) production [6,7].

Clinical manifestations of BTHS muscle abnormalities are evident from birth, presenting as myopathy, hypotonia, and delayed motor development [8,9,10]. Interestingly, BTHS skeletal muscle deficits are non-progressive and persist even following cardiac transplantation, confirming that peripheral myopathy is an autonomous tissue defect rather than a secondary consequence of cardiac insufficiency [11,12]. While cardiomyopathy and peripheral limb weakness are recognized hallmarks of the disease, the precise tissue-specific mechanisms governing survival-critical respiratory muscles remain poorly understood. Traditionally, clinical respiratory distress in BTHS patients (including dyspnea, orthopnea, and recurrent pneumonia) has been attributed to extrinsic factors such as congestive heart failure, neutropenia-driven lung infections, or systemic lactic acidosis [13,14,15]. Indeed, prevailing paradigms suggest that vital specialized muscles, including the bulbar musculature, extraocular muscles, and the diaphragm, are largely spared from the primary pathology [14,15,16,17]. However, the diaphragm occupies a distinct and demanding physiological niche, requiring a continuous combination of oxidative capacity for unceasing basal respiration alongside immediate glycolytic power for high-force behaviors like coughing. Because the diaphragm possesses a significantly higher mitochondrial volume density than peripheral limb muscles, it is theoretically highly vulnerable to TAZ deficiency and CL deficiency and subsequent cristae disorganization [18,19]. Whether the BTHS diaphragm and the associated pulmonary parenchyma are truly unaffected, intrinsically compromised, or capable of functional compensation remains a critical unaddressed question in BTHS pathophysiology.

In this study, we utilized a patient-tailored BTHS knock-in mouse model (*Taz^PM^*) harboring a clinical point mutation (*TAZ^D75H^*) within the critical TAZ acyltransferase domain [20]. This model successfully recapitulates stable mutant protein expression alongside severely diminished skeletal muscle CL levels [21]. Here, we demonstrate that juvenile and adult *Taz^PM^* diaphragms undergo a bifurcated compensatory remodeling response that sustains ventilation and prevents fatal respiratory failure under conditions of severe bioenergetic stress. This survival mechanism is orchestrated by the chronic activation of the mitochondrial Integrated Stress Response (ISR) via the Gcn2/eIF2α signaling pathway, which systematically halts global protein synthesis to conserve energy while selectively upregulating downstream defensive mitokines, metabolic sirtuins, and driving structural muscle remodeling. These adaptations reshape the *Taz^PM^* diaphragm into a highly specialized, slow-twitch motor system designed to preserve basal respiratory function at the cost of peak power development. Concurrently, we show that *Taz^PM^* lungs undergo highly specific metabolic reprogramming and organ-specific biochemical defense responses rather than generalized mitochondrial collapse or acute energy starvation. Together, these findings redefine our understanding of respiratory vulnerability in BTHS and uncover a novel, organ-specific survival program that preserves life-sustaining ventilation.

## 2. Materials and Methods

### 2.1. Mice

Wildtype (*wt*) and littermate *Tafazzin* (MGI:109626) *D75H* point mutant knock-in (*Taz^PM^*) mice were generated via breeding heterozygous *Taz^wt^*/*Taz^PM^* females with *wt* males (as *Taz^PM^* males are infertile), and PCR genotyping of resultant offspring was carried out as described [20]. Both juvenile (4-week-old) and young adult (6-month-old) male mice were examined, and *Taz^PM^* mutants were age-matched with their *wt* littermates for each and every assay. As BTHS is X-linked and predominantly affects males, this study exclusively examined male mice, as both growth restriction and myopathy were not present in female *Taz^PM^* mutants. It is unknown whether these findings are relevant for female *Taz^PM^* mice. Animal procedures and experimental conditions were refined to minimize harm to animals and performed with the approval of the Institutional Animal Care and Use Committee of Indiana University School of Medicine (IACUC approval #23095, approval date 13 July 2026).

### 2.2. Western Analysis

Juvenile and adult diaphragms and adult left and right inferior lung lobes (*n* = 3 per genotype/organ/age) were homogenized in RIPA protein lysis buffer (MilliporeSigma, Burlington, MA, USA) and ~30 μg sample/lane resolved using SDS-PAGE (Bio-Rad, Hercules, CA, USA) and transferred to PVDF Western blotting membranes, as described [20,21]. Following blocking in Blotting-Grade Blocker (Bio-Rad), blots were probed with primary and secondary antibodies, as well as a Total OxPhos Rodent WB Antibody Cocktail, to analyze the relative levels of OxPhos complexes (all listed in Table 1). To verify equal loading, all blots were subsequently stripped, washed, re-blocked, and then probed with housekeeping Gapdh or Tubulin (in Table 1) to monitor protein integrity and loading. Signals were detected using enhanced chemiluminescence substrate (SuperSignal West Pico PLUS Chemiluminescent and/or SuperSignal West Femto Maximum Sensitivity Substrate, ThermoFisher Scientific, Waltham, MA, USA), and images were collected on a Bio-Rad ChemiDoc. Arbitrary densitometry units were calculated and quantified using Prism software version 5.02 (GraphPad Software, San Diego, CA, USA), with signals normalized to the corresponding loading control and expressed relative to *wt* littermates.

### 2.3. Ultrastructural Analysis

For transmission electron microscopy (TEM), juvenile diaphragm and juvenile lung left and right inferior lobe samples were isolated and fixed in 2% paraformaldehyde and 2% glutaraldehyde fixative in 0.1 M cacodylate buffer (pH 7.2) overnight, processed, and imaged on a Phillips400 microscope (via IU EM Core) as described [20,21]. Mitochondrial morphology and grid counting to estimate mitochondrial number per 0.1 mm^2^ field (15 fields/organ/genotype and *n* = ~5–6/genotype/organ) were performed blinded to genotype and analyzed using ImageJ (version 1.54j).

### 2.4. Nucleotide Measurements

Micro-dissected juvenile and adult diaphragm and inferior lobe lung samples (*n* = ~3–5/genotype/organ) were immediately freeze-clamped in liquid nitrogen and stored at −80 °C until analysis. Frozen samples were homogenized in ice-cold 80% methanol/20% water using Kontes glass tissue grinders to remove protein. Ultra-performance liquid chromatography (UPLC) analyses were used to measure adenine nucleotides in the extracts with UV-Vis and mass spectrometry (MS) simultaneously, as described [20,21].

### 2.5. Histologic Analysis, Immunofluorescence Staining

For histological analysis, isolated diaphragms and left and right inferior lobes of the lung (*n* = 3/genotype/organ) were fixed in 4% paraformaldehyde overnight, dehydrated, and embedded in paraffin. Following serial sectioning (10 μm thickness), sections were stained with hematoxylin and eosin as described [20,21]. For immunofluorescence analysis, muscle fiber typing was performed as described [21]. Briefly, juvenile diaphragms (*n* = 3/genotype) were frozen in isopentane cooled in liquid nitrogen, cryosectioned (10 μm thickness), permeabilized, and probed via multiplex Type I myosin heavy chain (MHC), Type IIA MHC, Type IIB MHC, and Laminin antibody labeling as described [21]. Detailed primary and secondary antibody information is provided in Table 1. Images were analyzed for fiber type-specific cross-sectional area using the freely available software QuantiMus (version 1.3) [22].

### 2.6. PCR Array Analysis

Total RNA was isolated from juvenile diaphragms (*n* = 3/genotype) using RNA Spin Isolation columns (Qiagen, Germantown, MA, USA) following the manufacturer’s instructions. cDNA was synthesized using the RT2 First Strand Kit (Qiagen, Germantown, MA, USA) and qPCR was performed using the RT^2^ Profiler™ PCR Array Mouse Skeletal Muscle: Myogenesis & Myopathy (Qiagen). This array profiles 84 mouse genes (and controls) involved in skeletal muscle differentiation, myogenesis, and myopathy. Real-time PCR was performed as described [20] and raw data were analyzed using the 2^−ΔCt^ (Delta cycle threshold) method via the RT Profiler PCR Array Data Analysis (version 3.5).

### 2.7. Statistical Analysis

Data are presented as mean ± standard deviation, with *p* < 0.05 considered significant. Statistical analysis was performed via Prism software version 5.02 (GraphPad Software). Comparisons between the two groups, *wt* and *Taz^PM^* littermates, were performed by unpaired two-tailed *t*-test in all cases except for fiber typing, with statistical significance set at *p* < 0.05, *p* < 0.01, and *p* < 0.001. Comparisons of diaphragm fiber type distribution and fiber type-specific area were performed by two-way ANOVA followed by a Sidak post hoc test. PCR array *p* values were calculated based on a Student’s *t*-test of the replicate 2^−ΔCt^ (Delta CT) values for each gene in the triplicate *wt* and *Taz^PM^* littermate groups, with *p* < 0.05 considered significant.

## 3. Results

### 3.1. Taz^PM^ Diaphragmatic Skeletal Muscle Mitochondria Are Structurally and Functionally Abnormal

**Figure 1:**
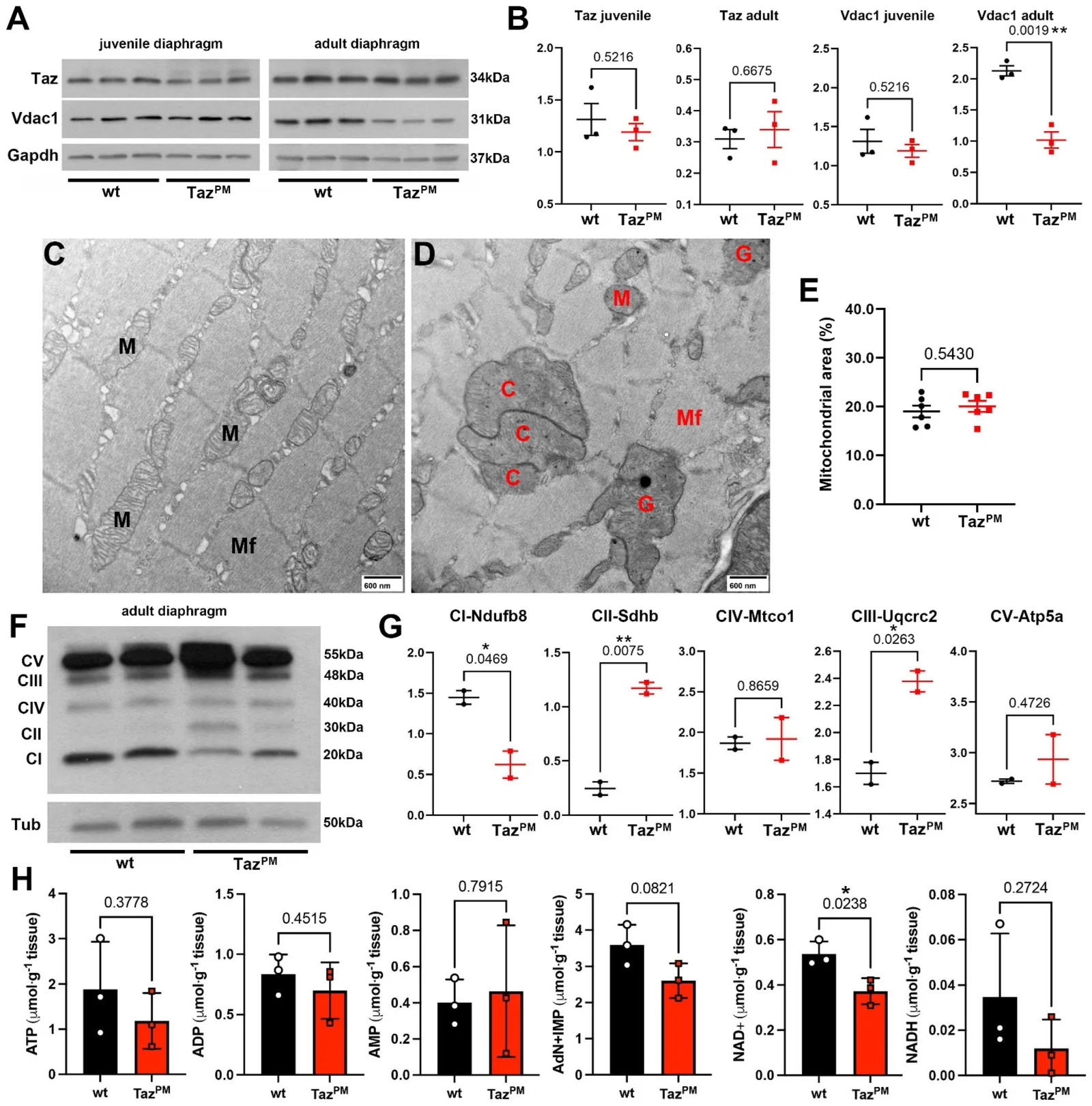
Dysmorphic mitochondrial structure and OxPhos function in Taz^PM^ diaphragms. (**A**,**B**) Western blot analysis of steady-state Tafazzin (Taz) and voltage-dependent anion channel-1 (Vdac1) protein levels in triplicate surviving juvenile (4-week-old) and adult (6-month-old) *wt*♂ and *Taz^PM^*♂ littermate diaphragms (*n* = 3/genotype), normalized to housekeeping control Gapdh levels (**A**). Quantification and statistical analysis of Taz and Vdac1 levels via two-tailed *t*-tests (**B**) in *wt*♂ (black) versus *Taz^PM^*♂ (red) littermate diaphragms. (**C**–**E**) Representative TEM images of juvenile diaphragms of *wt*♂ (**C**) and *Taz^PM^*♂ (**D**) littermate longitudinal sections (magnification 18,500×). Note that in contrast to *wt*♂ mitochondria that are filamentous and arranged in columns alongside sarcomeres, the *Taz^PM^*♂ mitochondria are disorganized and rounded, and several giant (indicated via red (**G**)) and/or clustered (indicated via red (**C**)) mitochondria are present (*n* = 6/genotype). Scale bars = 600 nm. Quantification of mitochondrial areas (**E**) as a % of diaphragm in *wt*♂ and *Taz^PM^*♂ littermates. (**F**,**G**) Western analysis of individual mitochondrial complex expression levels in duplicate adult (6-month-old) *wt*♂ and *Taz^PM^*♂ littermate diaphragms probed using an OxPhos rodent antibody cocktail kit (*n* = 2/genotype), with beta-Tubulin as the loading control. Quantification and statistical analysis revealed that *Taz^PM^*♂ CI (*) levels are statistically reduced, whilst both CII and CII are statistically increased compared to *wt*♂ littermates. (**H**) Ultra-performance liquid chromatography analysis revealed that ATP, ADP, AMP, total adenine nucleotides (AdN + IMP), and NADH levels each remain unchanged, but NAD+ levels are reduced (*p* = 0.05) in *Taz^PM^*♂ (red) versus *wt*♂ (black) littermate diaphragms (*n* = 3/genotype). Statistical significance was set at * *p* < 0.05, ** *p* < 0.01. Abbreviations: M, mitochondria; Mf, myofibrils.

As Taz is ubiquitously expressed in the IMM of all cells (apart from red blood cells [3]) and as we previously demonstrated that male *Taz^PM^* knockin mice express the mutant transacylase inactive Taz^D75H^ protein [20] at equivalent levels to *wt* Taz in hearts, gastrocnemius muscle, liver, bone marrow, and lymphocytes [20,21,23]; we examined Taz protein levels in both juvenile (4-week-old) and adult (6-month-old) *wt* vs. *Taz^PM^* isolated diaphragms. Significantly, surviving *Taz^PM^* express the mutant Taz^D75H^ protein at statistically equivalent levels to *wt* males in both juvenile and adult diaphragms (Figure 1A,B), phenocopying what is observed in our *TAZ^D75H^* patient lymphoblasts [23] and as reported in BTHS patients harboring diverse *TAZ* mutations [24,25,26,27]. To evaluate mitochondrial homeostasis and content, we quantified the outer mitochondrial membrane biomarker Vdac1, a key regulator of cellular energy metabolism [28]. Although there is an age-dependent change in mitochondrial abundance and/or mitochondrial protein expression accompanying maturation of the diaphragm and its energetic requirements, juvenile *wt* and *Taz^PM^* diaphragms expressed equivalent levels of Vdac1, whereas adult *Taz^PM^* diaphragms exhibited a significant reduction in Vdac1 expression compared to WT controls (*p* = 0.0019; Figure 1A,B). Because decreased adult Vdac1 expression implies a depressed respiratory capacity or perturbed metabolism, we investigated how the loss of Taz^D75H^ transacylase activity alters mitochondrial ultrastructure, oxidative phosphorylation (OxPhos) proteins, and levels of adenosine triphosphate (ATP)—the primary energy currency driving muscle contraction, active transport, and metabolic homeostasis [7,29,30].

Ultrastructural analysis via transmission electron microscopy (TEM) revealed severe mitochondrial abnormalities in surviving juvenile *Taz^PM^* diaphragms (Figure 1D). These mitochondria failed to form the interconnected reticular networks and organized columniform arrangements typical in *wt* diaphragmatic tissue (Figure 1C). Instead, juvenile *Taz^PM^* diaphragms displayed scattered, abnormally rounded mitochondria with irregular, thin cristae. Additionally, we observed a distinct population of enlarged megamitochondria and large mitochondrial clusters (Figure 1D) spanning roughly three to five times the size of *wt* mitochondria. Crucially, the total percentage of cellular areas occupied by mitochondria remained statistically comparable between juvenile *Taz^PM^* mice and *wt* littermates (Figure 1E). The emergence of mega- and clustered mitochondria likely represents an early structural hallmark of an adaptive or maladaptive mitochondrial stress response [31,32].

**Figure 2:**
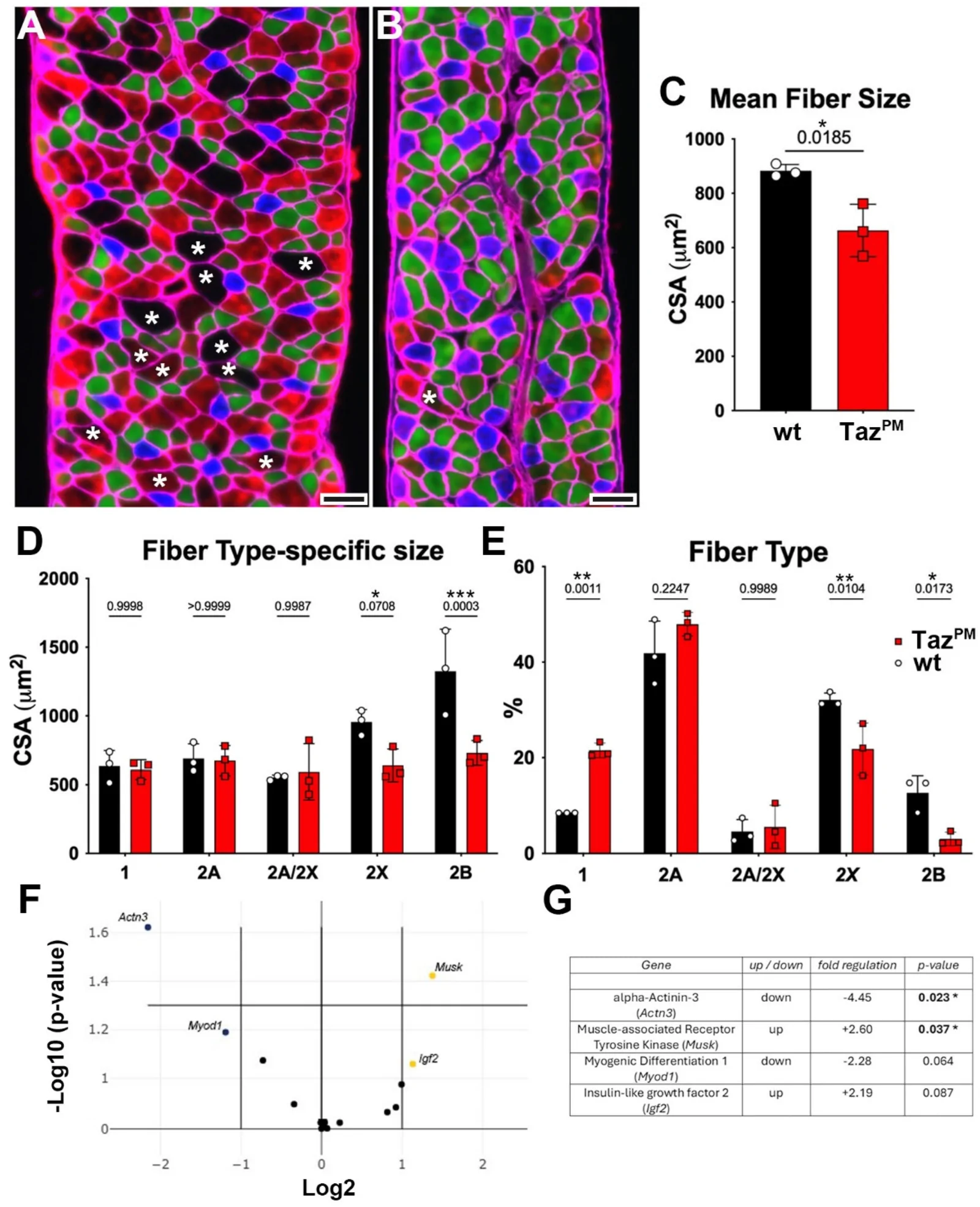
Skeletal muscle fibers are smaller and exhibit a muscle fiber type shift in *Taz^PM^*♂ mice. (**A,B**) Representative immunofluorescence for laminin (magenta), Type 1 MHC (blue), Type 2A MHC (green), and Type 2B MHC (red) was performed on juvenile *wt*♂ (**A**) and *Taz^PM^*♂ (**B**) littermate diaphragm muscle cross-sections (*n* = 3/genotype; magnification 630×). Unstained muscle fibers (indicated via *) are inferred to be Type 2× MHC (black). Scale bars = 100 µm. (**C**) Mean fiber type-specific cross-sectional area (CSA) and fiber size. (**D**) Frequency distribution of fiber size separated by MHC expression, and note there is a shift to smaller I/IIa fibers in *Taz^PM^*♂. (**E**) The proportion of fibers staining positive for each MHC. (**F**,**G**) Volcano plot (**F**) of juvenile *wt*♂ and *Taz^PM^*♂ littermate diaphragms differential steady-state mRNA level quantification of 84 key muscle differentiation, myogenesis, and myopathic effectors (*n* = 3/genotype). X-axis is log10 *p*-value, and the Y-axis is log2 fold differential expression levels. The table indicates candidates shown to be differentially expressed via PCR array analysis. Statistical significance was set at * *p* < 0.05, ** *p* < 0.01, *** *p* < 0.001.

Because TAZ is required for IMM cristae assembly and stabilization [4], and given the structural defects observed via TEM, we profiled individual electron transport chain (ETC) complexes embedded in the diaphragmatic IMM. Compared to *wt* littermates, adult *Taz^PM^* diaphragms demonstrated a complex ETC imbalance: Complex I (NADH:ubiquinone oxidoreductase subunit B8) trended down (*p* = 0.0469), while Complex II (Succinate dehydrogenase; *p* = 0.0075) and Complex III (Ubiquinol-cytochrome c reductase core protein 2; *p* = 0.0263) both trended upwards, consistent with adult Vdac1 (see Figure 2A,B) alteration. Conversely, both Complex IV (Cytochrome C oxidase I) and Complex V (ATP synthase alpha subunit) expression levels remained unchanged (Figure 1F,G). These stable Complex IV and V levels corroborate our TEM findings, indicating that overall *Taz^PM^* mitochondrial mass was likely not reduced despite structural remodeling. However, these assays were unable to verify if these protein Complexes integrate appropriately into the mutant IMM.

The primary function of the ETC is to generate an electrochemical proton gradient across the IMM, driven by the electron carrier nicotinamide adenine dinucleotide (NAD^+^), to sustain unceasing ATP synthesis during continuous breathing. Thus, we measured oxidized and reduced nicotinamide pools (NAD^+^/NADH) alongside adenine nucleotide profiles (ATP/ADP/AMP) to evaluate cellular redox state and energetic status [33]. Ultra-performance liquid chromatography (UPLC) revealed that adult *Taz^PM^* diaphragmatic high-energy phosphates (ATP, ADP, AMP, and AdN + IMP) and reduced NADH levels remained entirely unaffected compared to *wt* littermates (Figure 1H). However, adult *Taz^PM^* diaphragms displayed a significant depletion of oxidized NAD^+^ (*p* = 0.0238; Figure 1H), signaling a reduced total mitochondrial nicotinamide pool and an underlying energetic imbalance [33,34]. Taken together, these data demonstrate that *Taz^PM^* diaphragms undergo profound structural remodeling (cristae disorganization, megamitochondria, and clustering) and metabolic adaptation (ETC complex rebalancing and NAD^+^ depletion) required to preserve ATP homeostasis, sustain adequate ventilation, and support survival.

### 3.2. Taz^PM^ Diaphragm Exhibits a Fiber-Type Shift and Atrophy

To sustain the constant physiological demands of respiration alongside intermittent, high-force behaviors such as coughing or straining, the *wt* diaphragm is typically composed of an even mixture of highly fatigue-resistant slow-twitch and powerful fast-twitch muscle fibers. Diaphragmatic muscle strength is strongly correlated with myosin heavy chain (MHC) isoform expression, driven primarily by differences in the cross-sectional area (CSA) of distinct fiber types. Using immunofluorescence microscopy for MHC and laminin [21,22], we identified MHC Type 1, Type 2A, Type 2X, and Type 2B fibers within the diaphragmatic muscle of both *wt* and *Taz^PM^* adult mice (Figure 2). Significantly, *Taz^PM^* diaphragms exhibited a global reduction in myofiber size compared to *wt* littermates (*p* = 0.0185; Figure 2C), a morphological shift indicative of diminished maximal force-generating capacity. Crucially, this reduction in *Taz^PM^* CSA was not uniformly distributed across all fiber types. Instead, it was driven by a significant architectural shift toward smaller Type 2A fibers, compounded by the fact that the remaining Type 2x and 2b fibers were profoundly undersized (*p* = 0.070 and *p* = 0.0003, respectively; Figure 2D). These data indicate that *Taz^PM^* diaphragms undergo muscular atrophy and phenotypic remodeling. This shift toward smaller, fast-oxidative fiber types (Type 1 *p* = 0.0011; Figure 2E), coupled with a concurrent reduction in fast-twitch glycolytic fibers (Type 2x *p* = 0.0104, Type 2b *p* = 0.0173; Figure 2E), likely destines the muscle to a lower rate of force development. Because ATP consumption per amount of force is nearly 3 times greater in Type 2B fibers versus Type 1 fibers [35], the *Taz^PM^* diaphragm myofiber transition toward Type 1 fibers is likely an adaptive response that decreases energy use during breathing, making the diaphragms more energy efficient.

To reveal the molecular mechanisms underlying this *Taz^PM^* diaphragmatic pathology, we used a targeted qPCR array to profile a subset of 84 mRNAs critical to myopathy and myogenesis. Analysis of triplicate *wt* and *Taz^PM^* juvenile diaphragms via Volcano plot and the comparative 2^−ΔΔCt^ method revealed 4 enriched genes. Notably, only two genes achieved stringent statistical significance in the juvenile *Taz^PM^* diaphragm (Figure 2F,G): *α-Actinin-3* (*Actn3*), which was downregulated −4.45-fold (*p* = 0.023), and *Muscle-specific kinase* (*Musk*), which was upregulated +2.6-fold (*p* = 0.037). Actn3 is exclusively localized to the Z-discs of fast-twitch (Type 2) fibers to stabilize myofibrillar networks, and its marked reduction likely shifts fast-twitch fibers into a more oxidative and fatigue-resistant metabolic state. Conversely, the upregulation of Musk, a receptor tyrosine kinase essential for fast-twitch fiber maturation and neuromuscular junction maintenance [21], may induce ectopic acetylcholine receptor clustering within mutant diaphragms. Moreover, two additional regulators approached statistical significance (Figure 2F,G): *Myogenic differentiation-1* (*MyoD1*, −2.28-fold, *p* = 0.064) and *Insulin-like growth factor-2* (*Igf2*, +2.19-fold, *p* = 0.087). The depletion of MyoD1, a master myogenic regulatory factor upstream of Actn3 [36,37], could severely limit the regenerative pool of mature myofibers. Concurrently, elevated Igf2 levels, which typically drive localized muscle regeneration [38], may paradoxically downregulate downstream *Taz^PM^* structural myofiber genes and myocyte enhancers under chronic stress.

Together, the dysregulation of these effectors generates a conflicting intracellular signaling environment. Aberrant Igf2 and Musk expression promotes an abnormal state of over-activation and receptor remodeling, while the deficit in MyoD1 directly impedes the structural differentiation required to sustain growth. Simultaneously, loss of Actn3 forces surviving fast-twitch fibers into a more oxidative metabolic profile. Combined, these data demonstrate that in response to severe metabolic or energetic stress, the *Taz^PM^* diaphragm curbs its reliance on anaerobic glycolytic machinery (characterized by suppressed *Actn3* and *MyoD1*) and upregulates adaptive, compensatory pathways (*Musk* and *Igf2* signaling). This molecular remodeling ultimately transforms the *Taz^PM^* diaphragm from a rapid, powerful, yet fatigue-prone tissue into an assembly of smaller, slower, and fatigue-resistant oxidative fibers configured to preserve basal respiratory function.

### 3.3. Taz^PM^ Bifurcated Diaphragmatic Mitochondrial Myopathy

**Figure 3:**
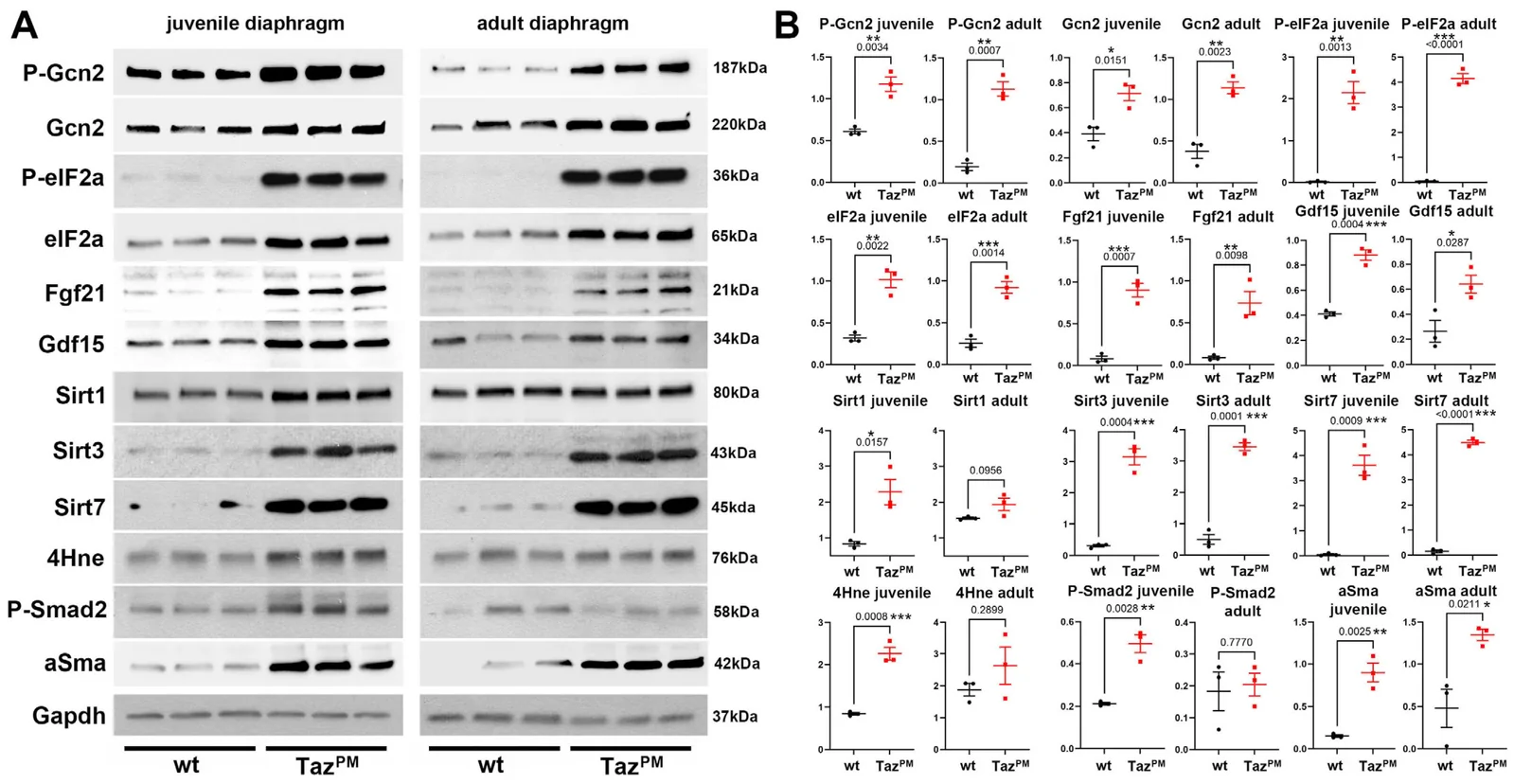
Bifurcated diaphragmatic mitochondrial myopathy in *Taz^PM^*♂ mice. (**A**) Western blot images of phosphoThr^899^-General control nonderepressible-2 (P-Gcn2), total General control nonderepressible-2 (Gcn2), phosphoSer^51^-eukaryotic Translation Initiation factor 2A (P-eIF2A), total eukaryotic Translation Initiation factor 2A (eIF2A), Fibroblast growth factor-21 (Fgf21), Growth differentiation factor-15 (Gdf15), Sirtuin-1 (Sirt1), Sirtuin-3 (Sirt3), Sirtuin-7 (Sirt7), 4-Hydroxynonenal (4Hne), phosphoSer^255^-Smad2 (P-Smad2), αSmooth muscle actin (aSma) steady-state expression levels in triplicate surviving juvenile (4-week-old) and adult (6-month-old) *wt*♂ and *Taz^PM^*♂ littermate diaphragms (*n* = 3/genotype), normalized to housekeeping control Gapdh levels. (**B**) Quantification and statistical analysis via two-tailed *t*-tests. Statistical significance was set at * *p* < 0.05, ** *p* < 0.01, *** *p* < 0.001.

To explore the signaling pathways triggering the observed mitochondrial remodeling and fiber-type shifts in *Taz^PM^* diaphragms, we investigated critical stress-responsive proteins. We previously demonstrated that adult *Taz^PM^* gastrocnemius limb muscles exhibit an elevated mitochondrial Integrated Stress Response (ISR), swollen and disorganized cristae, reduced Complex I Ndufb8 expression, as well as muscle fiber size reduction, fiber-type switching, and clustering [21]. Because the diaphragm typically contains 2 to 5-fold greater mitochondrial volume density than limb muscles [18], we initially examined General Control Nonderepressible 2 (Gcn2) and Eukaryotic Translation Initiation Factor 2A (eIF2α). These proteins act as key diaphragmatic stress sensors that protect against cellular stress and govern muscle adaptation in respiratory and chronic disease states [39]. Moreover, the phospho-eIF2α/total eIF2α ratio represents the gold-standard measurement for quantifying ISR activation [40,41]. Western blot analysis revealed that both juvenile and adult *Taz^PM^* diaphragms exhibit statistically significantly elevated phospho-Gcn2 (Thr899) and total Gcn2 levels, as well as phospho-eIF2α (Ser51) and total eIF2α levels (P-Gnc2 *p* = 0.0034 and 0.0007, Gcn2 *p* =001 and 0.0023, P-eIF2α 0.0013 and 0.0001, eIF2α *p* = 0.002 and 0.0014; Figure 3A,B). Once active, Gcn2 phosphorylates eIF2α, suppressing global protein synthesis while selectively upregulating stress-responsive genes to restore homeostasis [39,40,41]. Notably, concurrent increases in both total and phosphorylated forms of Gcn2 and eIF2α indicate that the *Taz^PM^* ISR is robustly sustained, with mutant diaphragms actively transcribing these stress effectors to maintain a prolonged response. Furthermore, the levels of the downstream, ISR-inducible hormones Fibroblast Growth Factor 21 (Fgf21) and Growth Differentiation Factor 15 (Gdf15) were statistically significantly elevated in both juvenile and adult *Taz^PM^* diaphragms (Fgf21 *p* = 0.0007 and 0.009, Gdf15 *p* = 0.0004 and 0.028; Figure 3A,B). Fgf21 and Gdf15 are “mitokines” that likely adapt energy expenditure, improve insulin sensitivity, and promote fatty acid oxidation [42] in response to the *Taz^PM^* mitochondrial ISR. Combined, these findings signify metabolic stress and robust, chronic activation of the ISR within *Taz^PM^* diaphragms.

Because we observed that *Taz^PM^* NAD+ levels were reduced (Figure 1H) and because NAD+-dependent deacetylase Sirtuins can act as metabolic energy sensors that are compensatorily upregulated via the ISR [43], we examined Sirtuin-1 (Sirt1), -3 (Sirt3), and -7 (Sirt7) expressions. Interestingly, Sirt1, Sirt3, and Sirt7 were all increased in juvenile *Taz^PM^* diaphragms (Sirt1 *p* = 0.015, Sirt3 *p* = 0.0004, Sirt7 *p* = 0.0009; Figure 3A,B), whereas only Sirt3 and Sirt7 remained elevated in adult *Taz^PM^* diaphragms (Sirt3 *p* = 0.0001, Sirt7 *p* = 0.0001; Figure 3A,B). Because Sirt1 and Sirt7 are elevated by nuclear/metabolic stress, and Sirt3 responds to mitochondrial stress and reactive oxygen species (ROS), this divergence reveals a continuous cellular attempt by *Taz^PM^* diaphragms to remodel their mitochondrial network, counteract energy imbalances, and boost antioxidant defenses. Correspondingly, the cytotoxic lipid peroxidation byproduct 4-Hydroxynonenal adductome (4Hne [44]) and phospho-Smad2-Ser255 marker that inhibits classic TGF-β signaling [45] were elevated in juvenile mutants (4Hne *p* = 0.0008, P-Smad2 *p* = 0.0028; Figure 3A,B) but remained unchanged in adult *Taz^PM^* diaphragms compared to *wt* littermates (Figure 3A,B). Conversely, alpha-Smooth Muscle Actin (αSma), a marker of active tissue fibrosis [46], was statistically significantly elevated in both juvenile and adult *Taz^PM^* diaphragms (*p* = 0.0025 and 0.021; Figure 3A,B). The combined excess of 4Hne, phospho-Smad2 (Ser255), and αSma in juvenile *Taz^PM^* diaphragms likely drives early mitochondrial dysfunction, oxidative stress, and myofibroblast activation. In contrast, the isolated elevation of αSma in adult mutants indicates progressive αSma upregulation and extracellular matrix remodeling. Combined, the accumulation of these stress effectors in the juvenile *Taz^PM^* diaphragm suggests an acute, global metabolic mitochondrial ISR compensatory remodeling response. Over time, the adult *Taz^PM^* diaphragm ceases to mis-express Sirt1, 4Hne, and phospho-Smad2-Ser255, shifting instead to a chronic, accommodated ISR state where structural mitochondrial and muscle fiber remodeling defects become stable.

### 3.4. Taz^PM^ Lung Mitochondria Are Abnormal, and Taz^PM^ Lungs Exhibit an Atypical Cellular Stress Response

Because the diaphragm operates in tandem with the lungs as a mechanical and metabolic partner, we examined whether *Taz^PM^* lungs exhibit associated structural, functional, or stress-related pathway changes. Although the lungs primarily function as gas-exchange organs, rather than mechanical thoracic cavity regulators, and they contain lower mitochondrial volume density than the diaphragm (~6% in lung versus 35% in diaphragm [47]), mitochondria can be heavily clustered within Type II alveolar epithelial cells (AT2 cells) and airway ciliated epithelial cells, which require substantial energy for specialized functions [48]. Because the left and right inferior lobes sit directly over the diaphragm and draw in the largest volume of fresh air during inhalation [49], we histologically, ultra-structurally, functionally, and molecularly compared *wt* versus *Taz^PM^* inferior lobes.

**Figure 4:**
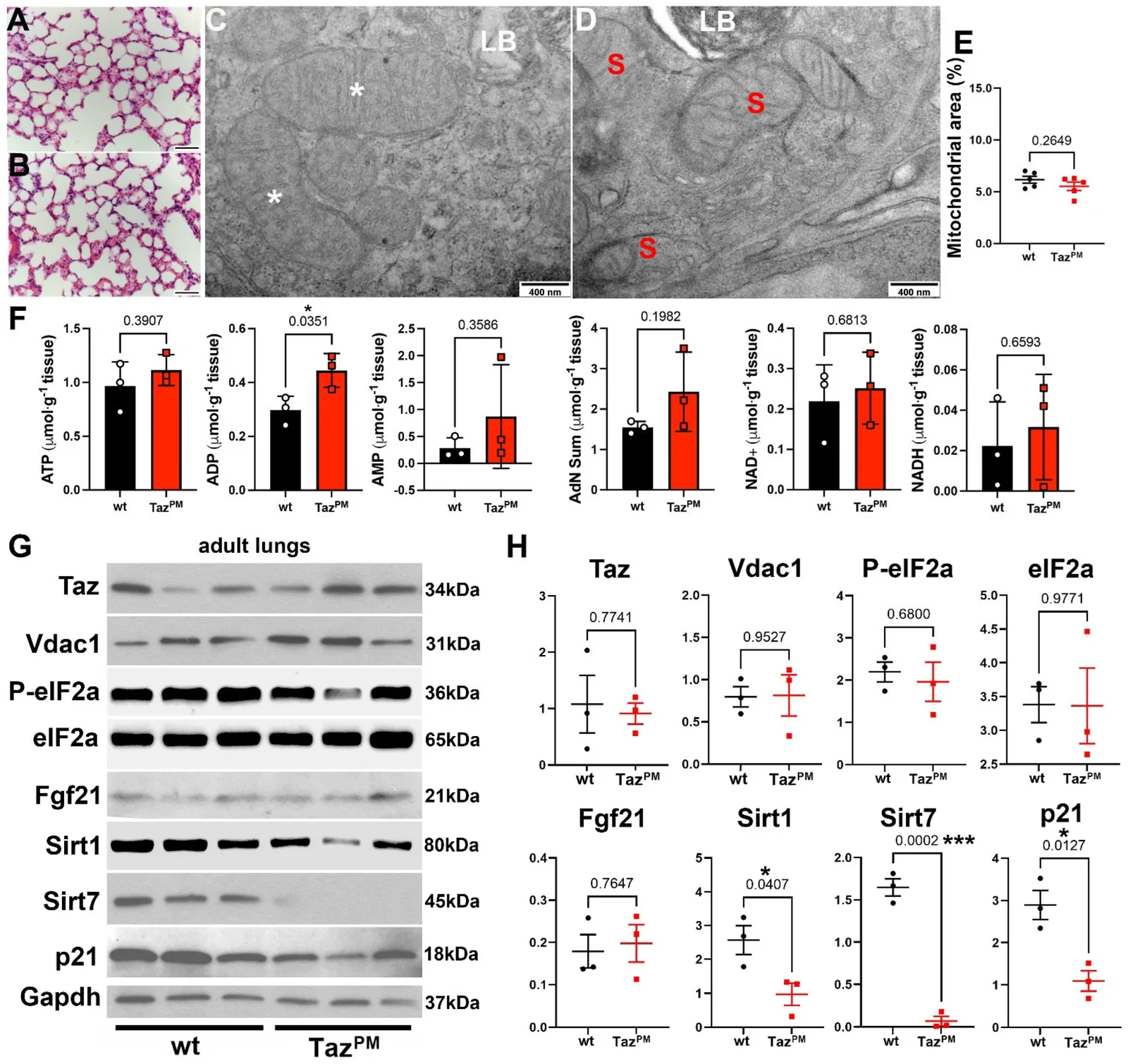
Lung abnormalities in Taz^PM^♂ mice. (**A**,**B**) Representative H&E histological sections (magnification 200×) of adult wt♂ and Taz^PM^♂ littermate inferior lobes (*n* = 3/genotype). Scale bars = 50 µm. (**C**–**E**) Representative TEM images (magnification 30,000×) of juvenile wt♂ (**C**) and Taz^PM^♂ (**D**) littermate inferior lung lobe Alveolar Type II cells (*n* = 5 inferior lobes/genotype). Note that in contrast to wt♂ mitochondria that contain compact, highly geometric, flat, sheet-like folds (indicated via *), the Taz^PM^♂ mitochondria cristae are disorganized, randomly oriented, with a swirling pattern (indicated via red S). Scale bars = 400 nm. Quantification of mitochondrial areas (**E**) as a % of inferior lung in wt♂ and Taz^PM^♂ littermates. (**F**) Ultra-performance liquid chromatography analysis revealed that ATP, AMP, total adenine nucleotides (AdN Sum), NAD+ and NADH levels each remain unchanged, but ADP levels are reduced (*p* = 0.05) in Taz^PM^♂ (red) versus wt♂ (black) littermate inferior lobes (*n* = 3/genotype). (**G**,**H**) Western blot images of Taz, Vdac1, eIF2A S^51^, total eIF2A, Fgf21, Sirt1, Sirt7, and p21 steady-state expression levels in triplicate surviving adult (6-month-old) wt♂ and Taz^PM^♂ littermate inferior lobes (**G**), normalized to housekeeping control Gapdh levels (*n* = 3/genotype). Quantification and statistical analysis via two-tailed *t*-tests (**H**). Statistical significance was set at * *p* < 0.05, *** *p* < 0.001. Abbreviations: M, mitochondria; LB, lamellar bodies.

Histology revealed that adult *Taz^PM^* littermate inferior lobes appear largely unaffected (Figure 4A,B). However, TEM analysis showed that mitochondria within juvenile *Taz^PM^* AT2 cells exhibit abnormal cristae structural phenotypes (Figure 4C,D). Specifically, while *wt* mitochondria contain compact, highly geometric, flat, sheet-like cristae folds, *Taz^PM^* mitochondrial cristae are disorganized, randomly oriented, and show a swirling pattern. This structural alteration may affect electron transport chain (ETC) supercomplex stability or abundance. Similar to *Taz^PM^* diaphragmatic mitochondria, the total percentage of the inferior lobe cellular area occupied by mitochondria remained statistically comparable between juvenile *Taz^PM^* mice and *wt* littermates (Figure 4E). UPLC analysis revealed that ATP, AMP, total adenine nucleotides (AdN Sum), NAD+, and NADH levels all remain unchanged, whereas ADP levels are statistically significantly increased (*p* = 0.0351; Figure 4F) in *Taz^PM^* inferior lobes. Increased ADP alongside stable ATP and AMP levels is a known hallmark of bioenergetic stress [7,50], suggesting that the swirling, disorganized *Taz^PM^* mitochondrial cristae are unable to support optimal OxPhos efficiency, prompting a subtle shift in inferior lobe nucleotide metabolism. To verify mitochondrial structural and membrane integrity, we examined Taz and Vdac1. Western blot analysis revealed that both Taz and Vdac1 levels remained unchanged in adult *Taz^PM^* inferior lobes compared to *wt* littermates (Figure 4G,H). This suggests that any metabolic shift occurs due to the altered Taz^D75H^ enzymatic activity or substrate availability rather than a change in total IMM Taz protein abundance or Vdac1-associated outer mitochondrial membrane pore anomalies. Furthermore, unlike *Taz^PM^* diaphragms (Figure 3), *Taz^PM^* inferior lobe P-eIF2α, total eIF2α, Fgf21, and Sirt1 levels were all unaltered. This indicates an absence of systemic stress sufficient to trigger a mitochondrial ISR, likely because global *Taz^PM^* ATP/AMP and NAD + /NADH pools remain stable. Moreover, Sirt7 levels are robustly reduced (*p* = 0.0002; Figure 4G,H), while p21 cell cycle inhibitor levels are also downregulated (*p* = 0.0127; Figure 4G,H) in *Taz^PM^* inferior lobes. The near-total absence of Sirt7 and reduced p21 may alter cell growth, cycling, and senescence pathways. However, both Gdf15 and Sirt3 were not detectable in *wt* or *TazPM* lungs. Combined, these results suggest that *Taz^PM^* inferior lobes exhibit localized metabolic reprogramming and organ-specific biochemical responses rather than generalized mitochondrial collapse or acute energy starvation.

## 4. Discussion

Uniquely, the *Taz^PM^* model carries a patient-derived point missense mutation (*TAZ^D75H^*) that preserves the structural integrity and abundance of the mutant protein but cannot properly enzymatically convert immature MLCL to mature CL [20]. Because the *TAZ^D75H^* mutation does not cause a protein-folding defect, it escapes degradation by cellular quality control pathways, resulting in stable Taz^D75H^ mutant protein levels despite the loss of enzymatic function. By utilizing the *Taz^PM^* knock-in mouse model, this study is able to decouple the structural consequences of CL dysfunction from simple protein deficiency and an absence of Taz’s scaffolding functions (as Taz physically associates ATP synthase and the ADP/ATP carrier complexes) in the IMM [51]. The structural and metabolic phenotypes exhibited in the *Taz^PM^* diaphragm reveal a complex, highly coordinated tissue-level adaptation to the absence of Taz enzymatic function and CL deficiency.

One of the most striking structural findings is the emergence of *Taz^PM^* giant megamitochondria and dense mitochondrial clusters, despite the total percentage of cellular area occupied by *Taz^PM^* mitochondria remaining unchanged (Figure 1D,E). This structural reorganization likely represents compromised mitochondrial fusion and impaired mitophagy, where *Taz^PM^* myocytes fail to clear away damaged or dysfunctional mitochondria [18,52], rather than simple swelling or alteration of the *Taz^PM^* mitochondrial biogenesis program. Indeed, megamitochondria and altered mitochondrial networks have been documented during ventilator-induced diaphragm dysfunction [53]. Direct assessment of mitochondrial dynamics and mitophagic flux will therefore be required to define the mechanisms underlying megamitochondrial formation in *Taz^PM^* diaphragm and to determine the relative contributions of impaired mitophagy and altered fusion/fission. At the biochemical level, this structural remodeling is accompanied by a stoichiometric imbalance within the mutant electron transport chain (ETC), characterized by a reduction in Complex I together with increased abundance of Complexes II and III (Figure 1F,G). This trend may reflect compensatory reorganization of the ETC in response to impaired Complex I stability or function, although appropriately powered studies incorporating direct measurements of respiratory flux will be required to establish the magnitude and reproducibility of the OxPhos defect. Defective respiratory supercomplex assembly is a well-established hallmark of BTHS [6,9,54], and Complex I may be particularly vulnerable to absent or dysfunctional *Taz^PM^* because its incorporation into functional respirasomes depends on an intact cardiolipin (CL) membrane environment [55]. Remarkably, despite severe cristae disorganization and an imbalanced ETC, high-energy adenylate pools (ATP, ADP, and AMP) remain completely stable in adult *Taz^PM^* diaphragms (Figure 1H). This compensatory, metabolic adaptation likely comes at a distinct redox cost, evidenced by the severe depletion of the oxidized NAD+ pool. The reduction in total cellular NAD^+^ points to an altered NAD^+^/NADH recycling efficiency [33], indicating that the *Taz^PM^* diaphragm is constantly operating near energetic exhaustion to preserve the basal ATP synthesis required to sustain unceasing ventilation. Future studies integrating mitochondrial morphology, content, respiratory function, and redox state within defined muscle fiber types will be important for determining whether these adaptations and their metabolic consequences differ across the heterogeneous fiber populations of the diaphragm. A second striking finding and in order to accommodate this fragile bioenergetic framework, the *Taz^PM^* diaphragm likely implements both a morphological and metabolic shift, transitioning from a high-power, mixed glycolytic/oxidative muscle into a smaller, slower, yet highly fatigue-resistant tissue. Since ATP use by a muscle fiber is dependent on fiber size and fiber type [35,56], the significant structural shift toward slow Type 1 and fast-oxidative Type 2A fibers and the severe atrophy of fast-twitch glycolytic Type 2X and 2B populations (Figure 2D) indicate ATP demand is decreased during mutant diaphragm contractions. This phenotypic shift toward energy-efficient, fatigue-resistant, oxidative fibers is likely triggered by *Taz^PM^* giant mitochondria [57] and via a distinct, conflicting transcriptional profile, namely robust downregulation of *Actn3* (Figure 2F,G). Because Actn3 is localized exclusively to the Z-discs of fast-twitch fibers to anchor glycolytic structural networks [58], its suppression destabilizes fast-twitch contractility, mechanically forcing myofibers to adopt a slower, more oxidative metabolic profile. This downstream shift may be initiated by the depletion of MyoD1, a master myogenic regulatory factor that directly drives *Actn3* transcription [37] and is responsive to CL metabolism [59]. Concurrently, the *Taz^PM^* diaphragm attempts to stimulate localized regeneration and maintain neuromuscular junction integrity by upregulating *Musk* and *Igf2* (Figure 2F,G). While intended to be compensatory, chronic activation of these pathways under sustained metabolic stress may generate a maladaptive intracellular signaling environment that maintains the *Taz^PM^* diaphragm in a persistently remodeled state, characterized by thinner, slow-twitch, oxidative myofibers biased toward energy-efficient, fatigue-resistant function. Although our fiber typing (myosin heavy chain) analyses demonstrate clear fiber-to-fiber differences, our energetics, Western blot, and mRNA analyses were performed on whole diaphragm homogenates, which do not allow analysis at the fiber level. Therefore, our energetics, Western blot, and mRNA analyses may underestimate or obscure the true changes that occur in any particular fiber type. Further, our data cannot distinguish whether the adult phenotype arises from impaired development of fatigable motor units, or from subsequent reorganization of these units after their development. Longitudinal studies across postnatal development, ideally incorporating motor-unit recruitment and fiber-type analyses, will therefore be required to distinguish between these mechanisms. Moreover, we recently demonstrated that *Taz^PM^* limb musculature can undergo severe atrophy accompanied by ISR activation in the absence of impaired OxPhos [21], raising the possibility that ISR activation may similarly contribute to *Taz^PM^* diaphragmatic atrophy through mechanisms such as mitochondria-induced proteostatic stress, acting as a non-mutually exclusive phenotypic driver. Although the present assays also cannot establish whether increased *Musk* reflects neuromuscular repair or remodeling, this finding is intriguing given that *Taz^PM^* limb muscles exhibit decreased Musk/Dok7, increased Chrna1 NMJ protein levels, and impaired NMJ integrity [21]. Functionally, the preferential preservation or relative predominance of oxidative Type I and IIa fibers, together with the marked reduction in the IIx/IIb population, may represent an adaptation that favors the sustained, fatigue-resistant activity required for eupnoeic breathing. Such remodeling could help preserve continuous ventilatory activity despite mitochondrial dysfunction but may simultaneously reduce the contractile and energetic reserve required for high-force, non-ventilatory behaviors such as coughing and other expulsive respiratory maneuvers. Importantly, an increased proportion of fibers normally associated with oxidative metabolism does not necessarily indicate preserved oxidative ATP-generating capacity in the setting of mitochondrial dysfunction. Direct measurements of fiber type-specific ATP production, energetic demand, and contractile performance will therefore be required to determine whether this remodeling represents an effective energetic adaptation or a compensatory phenotype that preserves basal ventilation at the expense of respiratory reserve. The signaling cascade driving this dual mitochondrial and myofibrillar transformation is governed by a robust, continuous activation of the Integrated Stress Response (ISR). The sustained phosphorylation of Gcn2 (Thr899) and eIF2α (Ser51) across both juvenile and adult *Taz^PM^* diaphragms establishes the ISR as a chronic response to the *Taz^D75H^* mutation, rather than a transient reaction to acute stress. Once activated, this axis selectively suppresses global translation to preserve ATP while driving the transcription of the critical stress-responsive mitokines Fgf21 and Gdf15 (Figure 3A,B). Meaningfully, FGF21 and GDF15 are elevated in BTHS patients’ plasma [60], and these hormones act locally to optimize fatty acid oxidation and adjust tissue-level energy expenditure [42] to match the diaphragm’s compromised respiratory capacity. Crucially, our data uncover a novel, bifurcated temporal dynamic within this chronic stress state as the *Taz^PM^* diaphragms mature from juvenile to adult (Figure 3A,B) and there is an age-dependent change in mitochondrial abundance and/or mitochondrial protein expression accompanying maturation of the diaphragm and its energetic requirements. The juvenile *Taz^PM^* diaphragm exhibits an acute, unstable metabolic crisis characterized by the significant elevation of Sirt1/2/3, the cytotoxic lipid peroxidation byproduct 4Hne, and inhibitory phospho-Smad2 (Ser255). The elevation of 4Hne demonstrates that the early structural remodeling of the IMM triggers significant ROS production and membrane lipid damage. Concurrently, the upregulation of phospho-Smad2 acts as an acute brake to inhibit classic TGF-β signaling [45], likely attempting to limit early fibrotic deposition during active tissue remodeling. However, as they reach adulthood, a state of stable accommodation is achieved. The normalization of 4Hne, Sirt1, and phospho-Smad2 in 6-month-old *Taz^PM^* diaphragms indicates that they have successfully managed the initial oxidative crisis. This stabilization is mediated in part by the persistent upregulation of the mitochondrial deacetylase Sirt3 and the nuclear sensor Sirt7 [61], which permanently bolster antioxidant defenses and optimize alternative metabolic pathways [43]. Nonetheless, this long-term metabolic accommodation comes at the expense of structural tissue quality and atrophy. While the acute oxidative markers normalize, αSma remains significantly elevated in both juvenile and adult mutant diaphragms. This persistent expression reveals progressive chronic remodeling of the *Taz^PM^* diaphragm extracellular matrix.

Gcn2 was investigated as an established nutrient- and metabolic-stress-responsive arm of the ISR [39] and, therefore, as a biologically plausible candidate given the pronounced mitochondrial and metabolic perturbations associated with Tafazzin enzymatic deficiency. The increased phosphorylation of Gcn2 (Thr899), together with elevated eIF2α phosphorylation (Ser51) and downstream ISR-associated responses, is consistent with engagement of the Gcn2–eIF2α signaling axis. However, these findings are associative and do not establish Gcn2 as the sole mediator of ISR activation. eIF2α is a convergent target of several stress-responsive kinases, including Gcn2, Hri, Perk, and Pkr, which are activated by overlapping but distinct cellular stressors [39,40,41]. Accordingly, future studies will be required to determine whether mitochondrial perturbations resulting from divergent *TAFAZZIN* mutations and/or CL deficiency also engage other eIF2α kinases, either independently of or in concert with Gcn2. Because the diaphragm operates in tandem with the lungs as a close functional partner during respiration, determining whether TAZ dysfunction extends to the pulmonary parenchyma is essential. Focusing on the inferior lobes, which handle the highest volumes of incoming fresh air [49], reveals an unexpected, organ-specific manifestation of the *Taz^PM^* mutation. In contrast to the mutant diaphragms, *Taz^PM^* lungs exhibit normally sized mitochondria but exhibit a transitional or remodeled state with randomly oriented cristae arranged in a distinct swirling pattern (Figure 4D). This specific phenotype points to a disruption in the physical assembly of IMM folds, which typically relies on the presence of CL [4,5,62]. Indeed, cristae are not static but are highly dynamic and frequently remodel in response to cellular stress, metabolic regimes, or physiological cues [63]. Despite these localized cristae geometric anomalies, the total percentage of inferior lobe cellular area occupied by *Taz^PM^* mitochondria remains statistically unchanged (Figure 4E). This mirrors the diaphragmatic *Taz^PM^* phenotype and confirms that total mitochondrial mass is preserved. Interestingly, this structural distortion results in a highly atypical metabolic profile that diverges from the classic, systemic mitochondrial collapse seen in *Taz^PM^* adult hearts [20]. UPLC analysis demonstrates that ATP, AMP, total adenine nucleotides, NAD+ and NADH levels all remain unaffected, yet ADP levels are statistically significantly increased (Figure 4F). This greater ADP amid stable ATP and AMP indicates an altered cellular bioenergetic state [7,50]. The swirling, disorganized cristae appear to undermine optimal oxidative phosphorylation (OxPhos) efficiency, triggering a subtle, localized shift in pulmonary nucleotide turnover rather than severe energy starvation. Western blot data confirm that total Taz^D75H^ protein abundance and Vdac1 outer-membrane pore levels are unaltered in adult *Taz^PM^* mutants (Figure 4G,H). This demonstrates that the bioenergetic shift stems purely from impaired Taz^D75H^ enzymatic activity or CL substrate scarcity rather than a drop in total IMM transporter mass or outer membrane anomalies. Remarkably, this localized pulmonary metabolic shift is completely uncoupled from the systemic, canonical stress pathways that dominate the *Taz^PM^* diaphragm. Unlike the muscular tissue, the inferior lobes show no alterations in P-eIF2α, total eIF2α, Fgf21, or Sirt1 levels. This confirms the absence of a broad, systemic mitochondrial ISR, likely because the global adenylate and nicotinamide pools remain adequately protected. Indeed, the *Taz^PM^* lung mounts a unique, organ-specific cellular response marked by a robust reduction in Sirt7 and a matching downregulation of the p21 cell cycle inhibitor (Figure 4G,H). Sirt7 is a crucial nuclear sirtuin tied to metabolic homeostasis, ribosomal RNA transcription, and stress survival [61,64]. Its near-total depletion, combined with suppressed p21, indicates that instead of initiating a mitochondrial stress pathway, the *Taz^PM^* pulmonary environment may alter its cellular growth, cell cycle progression, and senescence networks. This suggests that tissues with lower mechanical workloads may adapt to CL defects by altering cell-cycle progression and senescence networks rather than energy-conserving translational blocks.

Finally, as BTHS involves mitochondrial dysfunction and often leads to cardioskeletal myopathy and severe exercise intolerance [9,10,11,12,13], managing respiratory inflammation is crucial. Thus, BTHS patients are generally prescribed montelukast sodium to support an already fragile bioenergetic framework and block inflammatory leukotrienes in airways and alleviate further breathing stresses [65]. Indeed, our *TAZ^D75H^* patient is on preventative daily 10 mg of montelukast sodium therapy.

## 5. Conclusions

In conclusion, the *Taz^PM^* CL defect triggers a distinct, tissue-dependent adaptive dichotomy between mechanical and metabolic partners, which differs significantly from the canonical cardiac-specific and hindlimb-specific phenotypes observed in BTHS. *Taz^PM^* skeletal muscle adaptations represent a dynamic equilibrium between metabolic economy and functional demand. While diaphragmatic transition toward Type I and Type 2a MHC isoforms is typically an energy-conserving strategy, the maladaptive shift toward Type 2B fibers seen in *Taz^PM^* hindlimb muscles indicates a more complex failure to balance these needs. Thus, the diaphragm uniquely survives the loss of Taz transacylase activity through a profound, multi-layered adaptive response, sacrificing rapid contractile capacity (via *Actn3* and *MyoD1* suppression) and permanently restructuring its IMM network into megamitochondria to maintain a fragile, chronic ISR-mediated ATP homeostasis. In contrast, the adjacent lung inferior lobes avoid generalized mitochondrial collapse, systemic ISR activation, and energy starvation. Instead, the pulmonary tissue responds to CL-induced cristae swirling and altered ADP bioenergetics by executing an atypical, organ-specific signaling program defined by severe Sirt7 and p21 suppression. Together, these findings illustrate how the body leverages distinct regional strategies, ranging from muscular phenotype shifts to pulmonary cell cycle reprogramming, to maintain basal cardiorespiratory function and preserve survival.

## Figures and Tables

**Table 1:** Primary antibodies.

Antibody Name	Host Species	Source	Catalog Number	Dilution	Application
Laminin	Rabbit	Sigma-Aldrich, St. Louis, MO, USA	L9393	1:300	IF
Myosin Heavy Chain 1	Mouse	DSHB, Iowa City, IA, USA	BA-F8	1:100	IF
Myosin Heavy Chain 2A	Mouse	DSHB, Iowa City, IA, USA	SC-71	1:100	IF
Myosin Heavy Chain 2B	Mouse	DSHB, Iowa City, IA, USA	BF-F3	1:100	IF
α-SMA	Mouse	Sigma-Aldrich, St. Louis, MO, USA	A5228	1:20,000	WB
α-Tubulin	Mouse	Sigma-Aldrich, St. Louis, MO, USA	T5168	1:1000	WB
Cypher	Mouse	Santa Cruz, Dallas, TX, USA	SC-374359	1:1000	WB
eIF2A, phospho-Ser51	Rabbit	Abcam, Cambridge, UK	ab32157	1:2000	WB
eIF2A, total	Rabbit	Abcam, Cambridge, UK	ab169528	1:2500	WB
Gapdh	Mouse	Sigma-Aldrich, St. Louis, MO, USA	G8795	1:25,000	WB
Gcn2, phospho- Thr899	Rabbit	Invitrogen, Carlsbad, CA, USA	PA5-105886	1:10,000	WB
Gcn2, total	Rabbit	Cell Signaling	3302	1:1000	WB
4-Hne	Mouse	Invitrogen, Carlsbad, CA, USA	MA5-7570	1:1450	WB
Fgf21	Rabbit	GeneTex	GTX04856	1:1200	WB
Gdf15	Rabbit	Abcam, Cambridge, UK	ab105738	1:3000	WB
Musk	Rabbit	Invitrogen, Carlsbad, CA, USA	PA1-1741	1:400	WB
p21	Rabbit	Abcam, Cambridge, UK	ab188224	1:1000	WB
Sarcalumenin	Mouse	Invitrogen, Carlsbad, CA, USA	MA3-932	1:5000	WB
Smad2, phospho-Ser255	Rabbit	Abcam, Cambridge, UK	ab188334	1:5000	WB
Sirt1	Rabbit	Abcam, Cambridge, UK	ab12193	1:25,000	WB
Sirt3	Rabbit	Abcam, Cambridge, UK	ab246522	1:1650	WB
Sirt7	Rabbit	Abcam, Cambridge, UK	ab259968	1:5000	WB
Tafazzin	Mouse	Santa Cruz, Dallas, TX, USA	sc-365810	1:1000	WB
α-Tubulin	Mouse	Sigma-Aldrich, St. Louis, MO, USA	T5168	1:1000	WB
total OxPhos Rodent Antibody Cocktail	Mouse	Abcam, Cambridge, UK	ab110413	6 μg/mL	WB
VDAC1	Rabbit	Invitrogen, Carlsbad, CA, USA	PA1-954A	1:80,000	WB
Secondary antibodies					
Antibody Name	Host species	Source	Catalog number	Dilution	Application
Anti-Rabbit IgG (H + L)	Goat	Invitrogen, Carlsbad, CA, USA	A-21244	1:500	IF
Anti-Mouse IgG2b	Goat	Invitrogen, Carlsbad, CA, USA	A-21140	1:500	IF
Anti-Mouse IgG1	Goat	Invitrogen, Carlsbad, CA, USA	A-21121	1:500	IF
Anti-Mouse IgM	Goat	Invitrogen, Carlsbad, CA, USA	A-21045	1:500	IF
Anti-Mouse IgG2a	Goat	Invitrogen, Carlsbad, CA, USA	A-21143	1:500	IF
Anti-Rabbit IgG (H + L)	Goat	Bio-Rad, Carlsbad, CA, USA	170-6515	1:8000	WB
Anti-Mouse IgG (H + L)	Goat	Jackson Immuno Research, West Grove, PA, USA	115-035-146	1:8000	WB

DSHB = Developmental Studies Hybridoma Bank, IF = Immunofluorescence, WB = Western blot.

## Data Availability

The original contributions presented in this study are included in the article. Further inquiries can be directed to the corresponding author.
